# Leveraging the scientific findings to develop therapeutic strategies for dormant breast cancer cells

**DOI:** 10.18632/oncoscience.562

**Published:** 2022-09-13

**Authors:** Alejandra Ferrer, Yannick Kenfack, Andrew Petryna, Wadih Arap, Renata Pasqualini, Pranela Rameshwar

**Affiliations:** ^1^Department of Medicine-Hematology/Oncology, Rutgers New Jersey Medical School, Newark, NJ 07101, USA; ^2^Rutgers School of Graduate Studies at New Jersey Medical School, Newark, NJ 07101, USA; ^3^Rutgers Cancer Institute of New Jersey, Newark, NJ 07101, USA; ^4^Division of Cancer Biology, Department of Radiation Oncology, Rutgers New Jersey Medical School, Newark, NJ 07101, USA

**Keywords:** breast cancer, bone marrow, hematopoietic stem cells, cancer stem cells, gap junction

## Abstract

Breast cancer (BC) metastasis can occur decades before clinical diagnosis. During this time, the cancer cells (BCCs) can remain dormant for decades. This type of dormancy also occurs during remission where the dormant BCCs adapt cycling quiescence within the tissue microenvironment. BC shows preference for the bone marrow (BM), resulting in poor prognosis. The BM provides a challenge due to the complex niche between the peripheral interface and endosteum. The process of dormancy begins upon entry into the marrow with the changes facilitated through crosstalk between the cancer cells and tissue niche. More importantly, dormancy can occur at any time during the disease process, including the time during treatment. This perspective discusses the challenges posed by the marrow microenvironment to develop treatment. The article discusses the complex mechanisms at each compartment within the marrow niche and the added negative issue of toxicity to the endogenous stem cells.

Breast cancer (BC) remains a clinical issue, despite aggressive efforts for early diagnosis. There are ongoing efforts to close the gap on health disparity, specifically for the population living in the lower socioeconomic areas. Additionally, there are unwavering global research in basic and clinical science to develop new anti-cancer drugs while simultaneously training the next generation of scientists to address the gap in cancer biology [1]. A major challenge in BC treatment is the ability of the cancer cells (BCCs) to establish dormancy. The transition of BCCs into quiescent dormant cells is a continuous process, occurring years before clinical diagnosis and at any time during the disease process [2–8]. The preclinical establishment of dormancy could occur at least a decade. Thus, at diagnosis, it is highly likely that the cancer has already established. The dormant BCCs can be the source of tertiary metastasis during cancer resurgence, making it highly critical to understand the process of dormancy [9, 10].

This perspective focuses on dormant BCCs in the bone marrow (BM) since this organ is the preferred metastatic site for BC and is therefore a major source of tertiary metastatic disease [5, 11–13]. Furthermore, BC metastasis to the BM can lead to poor prognosis [13–15]. In cases where the BC is operable, the presence of BCCs in BM may predict cancer recurrence [15].

A major issue for targeting dormant BCCs is the similarity with healthy stem cells, including the hematopoietic stem cells (HSCs). Both types of cells express stem cell-associated genes and demonstrate similar function such as self-renewal and drug efflux [16]. Dormant BCCs can initiate tumor formation at tertiary metastasis and HSCs are needed to reconstitute the immune and blood systems [17–19]. Dormant BCCs have been categorized as CSCs, which resist current treatments and evade immune clearance [4, 13, 17, 20]. The discussed similarities between dormant BCCs/CSCs and healthy stem cells partly explain the challenges to target dormant BCCs in the BM as well as similar dormant BCCs in other organs. Specifically, healthy stem cells are located at metastatic tissues, which makes it difficult to target CSCs without untoward effects on the healthy resident stem cells. In the case of BM, HSCs share location with dormant BCCs close to the endosteum, which adds to the challenges of developing targeted therapy for residual BCCs in this organ [21, 22]. A cancer drug aimed to eliminate CSCs in the BM will be required to ensure safety of the HSCs. The tested drug would need to prevent BM ablation, which may lead to irreversible damage to the hematopoietic system with reduced immune and blood cells [23].

In order to develop efficient drugs, one must consider the different experimental approaches to evaluate their efficiency to recapitulate the tissue microenvironment where the cancer cells reside. It is yet to be determined if the same strategy could be used to target the cancer cells in the brain could be applied to cancer cells in the liver. This perspective will not present a comprehensive review of the various experimental methods. However, we will discuss the commonly used 2-dimensional (2D) cultures and provide evidence-based discussion on how this type of commonly used experimental approach should be complemented with 3D systems.

2D cultures of cancer cells and tumorspheres are common methods to screen drugs. The readouts of these methods for drug efficacy are mostly based on cell death and reduced number and size of tumorspheres [24]. Although these approaches are useful for high throughput screening, there are disadvantages when extrapolating the information to predict *in vivo* outcomes. Indeed, these methods could be an advantage when screening for target genes/network involved in cell autonomous mechanisms in the survival of CSCs [25]. Since a major mechanism by which BCCs survive is based on the cells’ interaction with niche cells such as resident BM cells. The tissue microenvironment regulates the behavior of BCCs, including their transition into dormancy [11, 21].

3-dimensional (3D) cultures is an advantage to include with 2D studies. 3D structures could be established by bioprinting or tissue organoids [26]. The BM, unlike other organs, is generally difficult to establish as an organoid or to be used in explant cultures. The advantage of 3D bioprinting is to design a structure that recapitulates the marrow [25]. The bioprinting can be established as a tissue with microenvironmental cells and molecules specific to the organ [26]. This would require coordinating how multiple cells and molecules are printed within a matrix that allows the survival of all cell types [26]. More importantly, 3D bioprinting could also mimic age since the stiffness of a microenvironment can be adjusted to the patient’s age. The incorporation of age into experimental models is important to compare cancer in young and aged individuals. Comparing the varied age groups would allow for the development of precise treatments, based on the behavior of the cancer cells within the particular aged tissue. This is fundamental to the field of precision/personalized cancer care as these studies will replace the current universal treatment, regardless of the patient’s age. Despite the advantages of bioprinting, there are weaknesses in this model when considering the complex cellular components of an organ, in this case, BM. In this regard, parallel use of 3D bioprinting would benefit with research using organoids as well as mouse model such as patient derived xenografts (PDX).

The BM niche includes several cell types such as fibroblasts, macrophages and mesenchymal stem cells (MSCs) and CXCL12 abundant reticular cells (CAR cells), create a complex microenvironment that support the behavior of BCCs including transition into dormant cells to evade treatment [18, 21, 27–29]. The mechanisms by which BM niche cells facility dormancy are complex and includes direct and indirect intercellular interaction. Direct intercellular interaction occurs through gap junction (GJIC) between cells of the BM microenvironment and CSCs to acquire cellular quiescence [14, 30, 31]. Indirect interaction between BCCs and BM niche cells can occur by soluble and insoluble secretome such as cytokines and microvesicles [11, 20, 32].

GJIC between MSCs and CSCs is of tremendous benefit to BCCs since MSCs, through the production of TGFβ1, can increase immune suppressive regulatory T-cells while decreasing cytotoxic natural killer cell activity [14, 29]. MSCs undergoing senescence could mediate BCC invasion, perhaps through their differentiation into carcinoma-associated fibroblasts [33, 34].

The tissue microenvironment has a key role in directing the behavior of BCCs including transition to CSCs. As discussed above, after transition to CSCs, the challenges to target these cells are amplified. Insights in the interaction between tissue microenvironment and BCCs from the time of entry into BM to the endosteal niche where the cells are in cellular dormancy will provide insights into how BCCs are able to survive for decades without detection [10, 13]. An understanding by which the tissue niche maintains BCCs in cellular quiescence will simultaneously provide information on the mechanisms of reverse dormancy. Specifically, when the dormant CSCs transition into metastatic cells during cancer recurrence. It is important to have indepth dissection of stepwise processes since this will lead to safe and efficacious treatment to quickly eliminate existing dormant BCCs and to prevent dedifferentiation of non-CSCs to CSCs [32].

To reiterate, experimental model systems are key to understanding how BCCs behave within different tissue microenvironments. In order to target dormant BCCs within their niche, one must consider how to reverse the current dormant BCCs for safe treatment. This outcome is likely to require treatment with different drug combinations, perhaps by repurposing of existing drugs. Since CSCs have been shown to form gap junctional interaction (GJIC), in addition to other types of interaction with BM niche cells, breaking these interactions are obvious approaches [27, 30, 31]. Reversing dormancy through breaking the intercellular interactions are likely to induce the dormant CSCs into differentiated cycling cells, which will make them drug targets with the class of drugs that require cell cycle [35]. Exiting cell quiescence and stemness are likely to induce differentiation, which could be directed with differentiating agents. As an example, NFĸB, which has been shown to maintain stemness, could be targeted with proteasome inhibitors such as bortezomib or carfilzomib to differentiate CSCs [36]. Similar to these findings in hematological malignancies, this approach could allow for the otherwise dormant CSCs from BC to induce sensitivity to other drugs such as DNA alkylating agents [30, 36].

The approach discussed in the previous paragraph will require studies by a team with varied scientific background. Reverse dormancy, if not treated properly, could lead to overt metastasis including cancer growth in the brain [30]. Indeed, the scientific community has acknowledged the need to reverse dormancy while others believe that strategy should be developed to maintain BCCs in dormancy [27, 37]. However, there are uncontrolled events that could reverse dormancy as noted during tumor recurrence. An example of unforeseen BC recurrence could be explained by infection when the microbiome products could interact with Toll receptors on dormant BCCs to induce metastasis [27]. Another unforeseen method could occur when the hypothalamus pituitary axis is activated by psychological stress, resulting in increased hormones that could influence BCC growth as well as suppress the immune system to provide an advantage to the tumor cells [38, 39].

The process of BCCs towards dormancy is highly complex and includes multiple factors and the specific BM niche. Thus, the question is how to develop targets to reverse and prevent dormancy without overt toxicity that might be irreversible. As indicated above, the entry of BCCs as dormant cells is a continuous process. Thus, in order to get a clear understanding of the mechanisms by which BCCs begin the transition into dormancy at the interface of the peripheral vascular system, it is prudent to focus at the interface between the periphery and marrow cavity [32]. Studies on the stepwise processes by which the incoming BCCs interact with MSCs that are in contact with the blood vessel (Figure 1). MSCs have different roles in protecting BCCs to resist drugs [14, 21, 29]. The question is how BCCs take advantage of the BM microenvironment at the interface to begin transition as dormant cells? The experimental evidence indicated that the initial interaction between BCCs and MSCs occur when extracellular vesicles/exosomes (EVs) released from MSCs enter BCCs to initiate cycling quiescence [32]. In a delayed process when the BCCs are able to communicate with MSCs, the MV cargo changed, resulting in rapid dedifferentiation of the BCCs into CSCs.

**Figure 1:**
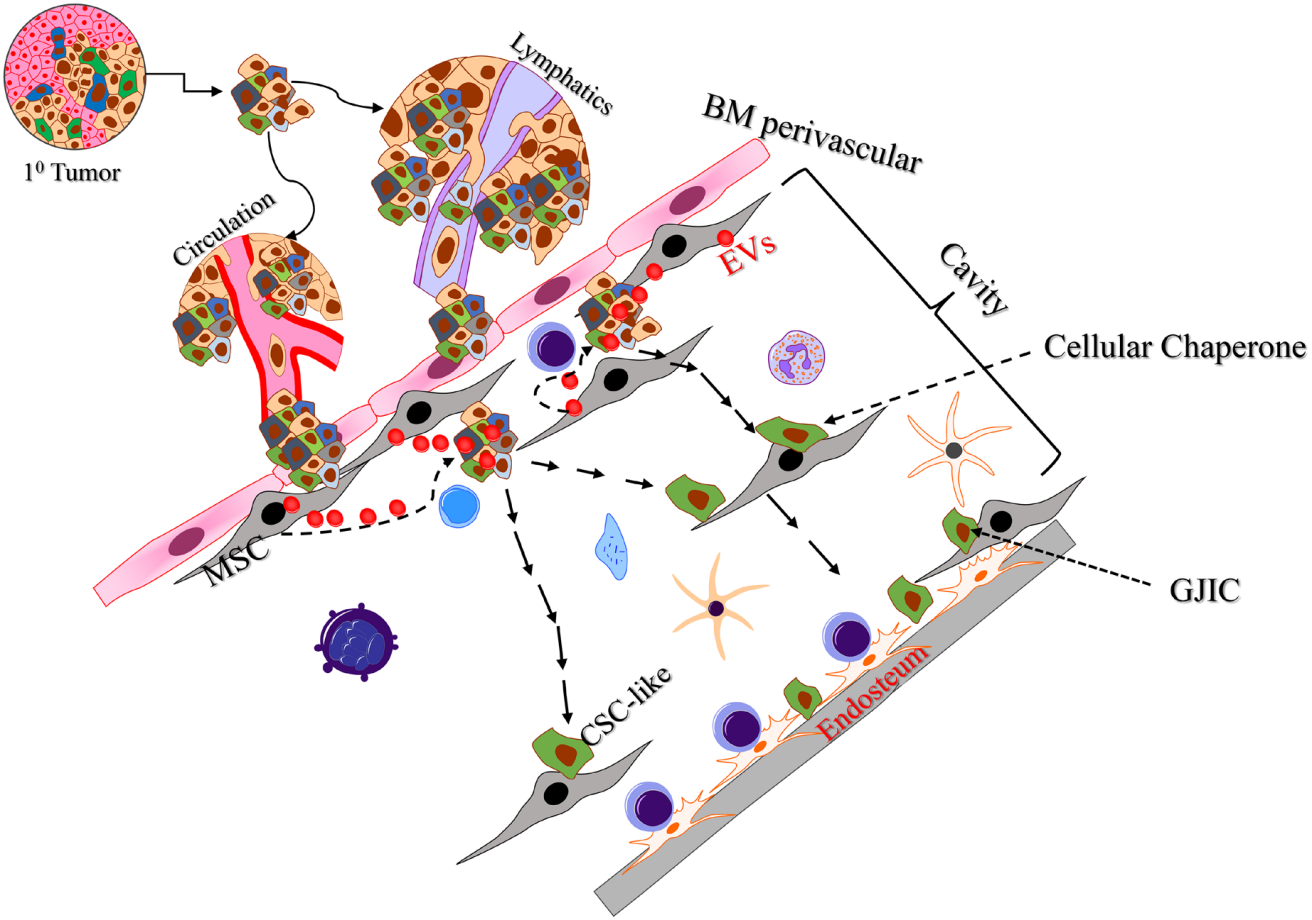
Steps towards BCCs transitioning dormant cells. Shown are BCCs entering the marrow, contacting the MSCs. Shown are MVs entering the entering BCCs. The dedifferentiated BCCs are shown interacting with MSCs (cellular chaperone). The final movement ended at the endosteal region where the dedifferentiated cells form GJIC with stromal cells. Abbreviations: BM: Bone Marrow; CSC: Cancer Stem Cells; GJIC: Gap junctional intercellular communication; MSCs: Mesenchymal Stem Cells.

Exosomes are part of the family of EVs involved in BC dedifferentiation, briefly discussed above [32]. The EV size described above averaged 100 nm in diameter and expressed endosomal markers. The role of RNA in EVs are described [32]. However, other EV cargos such as lipids and proteins could have roles in the dedifferentiation process [40, 41]. EVs as a messenger of dedifferentiation is not a surprise because these vesicles can regulate related cancer cell behavior [21, 42]. Single cell sequencing of BCCs exposed to the initial set of EVs and the late released EVs coming from MSCs, showed distinct differences in the resulting BCC population [32]. The stepwise process prepared the BCCs for dormancy by responding to the MSC-derived EVs by transitioning into cell cycle quiescence [32]. This first step, which involved epigenetic changes, responded to other MSC-derived EVs with different cargo to change the transitioning BCCs into a more homogenous population of stemness [32]. Extrapolating these findings to a physiological setting in a patient with active disease, it would appear that a single drug is unable to target the vast subset of BCCs, which would be undergoing a dynamic molecular change within the microenvironment.

There is a need for robust research on induced epigenetic changes as BCCs transition into dormant CSCs. There is a strong likelihood of a link between cell fate changes in BCCs and reorganization of the epigenome. The expression of TET enzymes and TDG in BCCs depended on early or late exposure to EVs [32]. TDG excision of TET-mediated DNA oxidations (5fC and 5caC) allows BER to impart DNA demethylation. Thus, up-regulated TET2 and TDG was significant to the dedifferentiation process, which is consistent with TET2-mediated DNA demethylation in somatic cellular reprograming, stem cell maintenance, and cancer cell quiescence [43, 44]. Thus, since TET2 mRNA is enriched in the naïve EVs, which causes the initial steps in BCC dedifferentiation, we propose a link between TET2-mediated DNA demethylation and dedifferentiation for the acquisition of stem cell signatures. These exciting findings will form the basis for future studies on indepth genome wide changes as BCCs respond to the complex BM microenvironment to undergo dormancy.

Future research is required with different size of EVs, which might carry distinct cargo and the outcome of dedifferentiation could be the result of adding different types of EVs [41, 45, 46]. Another limitation in the role of EVs is the lack of data on triple positive BC. The information on triple negative BCCs is an advantage since this type of cancer is prevalent in underrepresented minority without targeted treatment.

It was interesting to note that the dedifferentiated BCCs at the peripheral-marrow interface established a cellular chaperone relationship with MSCs [32]. The cellular complex migrate to the endosteal region where the dedifferentiated BCCs are able to establish long-term cell quiescence as dormant CSCs [16, 32, 47]. The observed contact with MSCs throughout the marrow cavity could offer different protection to the dormant BCCs. Firstly, as the BM is a primary lymphoid organ, there should be an immune surveillance system to eliminate the dormant BCCs. However, close contact with MSCs would allow for the increase of regulatory T-cells to protect the dormant BCCs [14, 29]. Secondly, these MSCs can differentiate into fibroblasts, which are part of the BM stromal cells where the dormant BCCs can establish GJIC [16, 31]. In addition to the early events shown for MSC with respect to dormancy, MSCs could be active in sustained dormancy at the later stage due to their location throughout the marrow.

An interesting finding that should not be ignored is the identification of genes similar those in CSCs and other stem cells in the more differentiated BCCs, referred as Oct4a^lo^ BCCs [32, 48]. One can argue that cancer has adapted the properties of stem cell. However, only the more differentiated populated of BCCs shared these similar genes. Indeed, this same population was showed to dedifferentiate relatively easy as compared to the other BCC subset [32]. The scientists involved in drug development might want to consider this finding as a framework because the Oct4^lo^ BCC subset are cycling cells and could be drug sensitive when studied independently. This underscores the need for 3D models, discussed above, even in drug screening.

GJIC between MSCs and CSCs might lend to sharing of molecules through the connexin channels [14]. MSCs can maintain dormancy close to the endosteum by supporting gap junction between M2 macrophage and CSCs [27]. Overall, the findings, when combined with other reports, indicate a key role for MSCs during early and late stage dormancy. However, the question is how to break the junction safely when connexin 43 (Cx43) is needed by the hematopoietic system [49]. N-cadherin has been shown to be needed for Cx43 to migrate to the membrane and to regulate its expression could be a relevant target, perhaps examining the linked γ-secretase [30].

Overall, this perspective highlight the complex mechanism by which the marrow niche can hinder treatment of BC. The involvement of drug resistance and immune evasion is not limited to cells but to EVs. The discussed perspectives could be extrapolated to other metastatic sites and to the establishment of experimental models. A critical thought is to determine how different sources of MSCs could be used to deliver drugs and RNA, even to brain [21]. It is clear that MSCs have memory to home to the originating organ/tissue. Research is needed to evaluate the efficacy of using particular source of MSCs. The investment of such cells in drug delivery is an advantage as these cells can cross allogeneic barriers and can be available as off-the-shelf source during treatment. The experimental data showed a role for the Wnt-β-catenin pathway in EV-mediated dedifferentiation [32]. Although the Wnt pathway has been shown to be activated in triple negative BCCs, there is a seeming novel role for this pathway during dedifferentiation of BCCs into CSCs [32, 50]. Furthermore, VerElect algorithm scored the catenin pathway high for known BC genes [32]. Based on the discussion on potential treatment, there are drugs that can target the γ-secretase and Wnt pathways that could be repurposed for BC targeting to prevent and perhaps treat dormant BCCs.
